# Protein GB1 Folding and Assembly from Structural Elements

**DOI:** 10.3390/ijms10041552

**Published:** 2009-04-08

**Authors:** Mikael C. Bauer, Wei-Feng Xue, Sara Linse

**Affiliations:** 1 Department of Biophysical Chemistry, Lund University, Chemical Center, SE-22100 Lund, Sweden; 2 Present address: Astbury Centre for Structural Molecular Biology, University of Leeds, LS2 9JT, Leeds, UK; E-Mail: w.f.xue@leeds.ac.uk (W.F.X.)

**Keywords:** Protein folding, protein reconstitution, fragment complementation, prefolding, electrostatic interactions, salt screening

## Abstract

Folding of the Protein G B1 domain (PGB1) shifts with increasing salt concentration from a cooperative assembly of inherently unstructured subdomains to an assembly of partly pre-folded structures. The salt-dependence of pre-folding contributes to the stability minimum observed at physiological salt conditions. Our conclusions are based on a study in which the reconstitution of PGB1 from two fragments was studied as a function of salt concentrations and temperature using circular dichroism spectroscopy. Salt was found to induce an increase in β-hairpin structure for the C-terminal fragment (residues 41 – 56), whereas no major salt effect on structure was observed for the isolated N-terminal fragment (residues 1 – 41). In line with the increasing evidence on the interrelation between fragment complementation and stability of the corresponding intact protein, we also find that salt effects on reconstitution can be predicted from salt dependence of the stability of the intact protein. Our data show that our variant (which has the mutations T2Q, N8D, N37D and reconstitutes in a manner similar to the wild type) displays the lowest equilibrium association constant around physiological salt concentration, with higher affinity observed both at lower and higher salt concentration. This corroborates the salt effects on the stability towards denaturation of the intact protein, for which the stability at physiological salt is lower compared to both lower and higher salt concentrations. Hence we conclude that reconstitution reports on molecular factors that govern the native states of proteins.

## 1. Introduction

Non-covalent interactions within and between proteins govern many biological processes involving folding and binding reactions. Folding reactions are inherently difficult to study due to their unimolecular nature, as in many cases their equilibrium constants are high enough to yield very low populations of the unfolded state, independent of protein concentration. Binding reactions on the other hand, due to their bimolecular nature, allows dilution of the system to a regime where all states are significantly populated and straightforward to quantify. Fragment complementation, the reconstitution of a correctly folded and functional protein from separate fragments [1–5], is a binding process governed by interactions of non-covalent character under the restrictions imposed by the covalent backbone. Fragment complementation of proteins thus presents an attractive alternative approach to protein folding studies and can be used as an approach for studying the relative contributions to protein assembly from different kinds of non-covalent interactions, such as hydrogen bonding, electrostatic, hydrophobic and van der Waals’ interactions. While the relative contributions of these interactions to protein folding has to be obtained from extrapolation from very harsh and non-physiological conditions, the reconstitution reaction can be studied directly at the conditions of interest, including physiological conditions and the conditions under which the corresponding intact protein is maximally stable.

As stated above, an inherent limitation with equilibrium folding studies is the very low population of the unfolded state under physiological or similar conditions. The contribution of different kinds of interactions to the free energy of folding thus have to be obtained by very long extrapolations from conditions where the relative values of these interactions are largely perturbed. Protein reconstitution circumvents this obstacle as the system can be diluted down to a concentration regime where both the bound and free states are significantly populated. Hence equilibrium parameters can be determined by high precision directly at the condition of interest. Recent fragment complementation studies have shown that interactions involving the hydrophobic core residues of proteins are by far more important for favoring protein assembly than interactions involving charged residues [6–10]. The modification of a single hydrophobic core residue into a smaller hydrophobic one can change the affinity between the two protein fragments by as much as four orders of magnitude [6]. Corresponding numbers for charged residues are around one order of magnitude [7,8]. Based on the observed linear correlation between effects on the free energy of protein assembly from fragments and on the free energy of folding of the corresponding intact chain [6,7,10], we can conclude from these studies that interactions involving the hydrophobic core residues are by far more important for protein stability and folding than interactions involving charged residues.

The so called Levinthal paradox states that folding of a protein would not be completed within the lifetime of the universe if it was a random process through all possible conformations of the polypeptide. For folding to work on the biological time scale, the degrees of freedom of the polypeptide chain must be limited at an early stage. Several protein folding models, incorporating early states with limited degrees of freedom, have been proposed e.g. the hydrophobic collapse model [11], the nucleation model [12] and hierarchical processes [12,13]. Recent Monte Carlo simulations have shown that a sufficient criterion for folding to proceed at high rate and fidelity to the native state is that productive (native) contacts on average survive longer than non-productive (non-native) contacts [14]. For many proteins, it has been found that local building blocks are initially formed and then interact to make up the complete tertiary fold, resembling fragment complementation [12] with the important restriction that protein folding is a unimolecular reaction while reconstitution is a bimolecular reaction.

**Figure 1.**
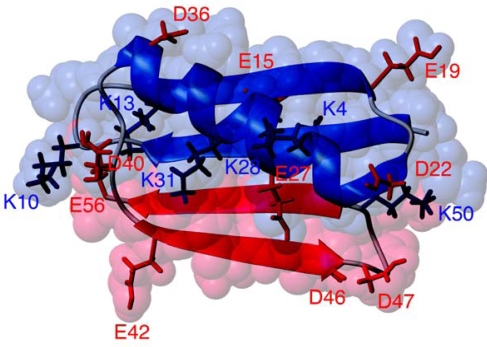
The structure of PGB1 with residues 1 – 40 in blue and 41 – 56 in red with the backbone shown as a ribbon diagram on top of the space-filling model. Charged sidechains are shown as sticks with Asp and Glu side chains in red and Lys side-chains in dark blue. The figure was prepared from PDB file 2GB1 using MolMol software [35].

The present reconstitution study concerns the 56 amino acid immunoglobulin G-binding domain 1 of Streptococcal Protein G (PGB1). Fragment complementation has been observed in many cases with high affinity complexes of polypeptide fragments reconstituting the native fold and yielding similar functional characteristics as the corresponding intact protein. Reported examples of reconstituted proteins belong to many structural and functional classes and include thioredoxin [15], cytochrome *c* [16], dihydrofolate reductase [17], ribonuclease A [1,2,18], staphylococcal nuclease [19], troponin C [20], calmodulin [21], calbindin D9k [6,22], calbindin D28k [23], calretinin [24], protein GB1 [25], the chymotrypsin inhibitor-2 [26], ubiquitin [27] and monellin [28]. For reviews see references [3–5]. In the case of PGB1, two fragments encompassing amino acid 1 – 40 and 41 – 56, have previously been reported to reconstitute with an equilibrium dissociation constant in the micromolar range [25]. PGB1, as shown in Figure 1, has a β-grasp fold consisting of an α-helix packed on a four-stranded β-sheet [29,30]. The smaller of the two fragments has an interesting property in isolation. Although it is only 16 amino acids long, it has a tendency to form a native-like β-hairpin structure [31,32]. This makes it one of the smallest known peptides to fold into a defined structure and it is a popular target for folding simulations and experiments [33,34].

Previously, the pH- and salt-dependent stability of an intact PGB1 variant has been thoroughly measured [36]. The variant, named PGB1-QDD, contains the mutations T2Q, N8D and N37D in order to avoid N-terminal processing and deamidation. Lower stability towards denaturation of PGB1-QDD was seen at 150 mM NaCl compared to 0 or 2 M NaCl, over a wide pH range [36]. Here we have studied the thermodynamics of the reconstitution of PGB1-QDD and the effects of salt concentrations and temperature on the reconstitution process. The affinity between PGB1-QDD fragments is measured as a function of salt and temperature using circular dichroism spectroscopy. The results yield valuable clues to the non-covalent factors that govern the assembly of PGB1 and indicate a switch in folding behavior as a function of salt concentration.

## 2. Results and Discussion

### 2.1. Salt dependence of CD spectra for fragment mixtures

**Figure 2.**
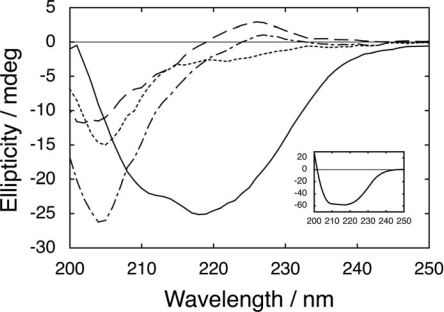
Far-UV CD spectra of 584 μM N41-QDD (dashed line), 584 μM C16 (dotted line), and an equimolar mixture of N41-QDD and C16 (584 μM each, solid line). The sum of the N41-QDD and C16 signals (dash-dotted line). The insert shows the spectrum of 584 μM intact PGB1-QDD. All samples are in 5 mM sodium phosphate buffer pH 5.5 at 278 K.

The interaction between two fragments of PGB1-QDD comprising residues 1 – 41 (N41-QDD) and 41 – 56 (C16), respectively, was studied using CD spectroscopy at four salt concentrations (0, 0.15, 0.5 and 2 M added NaCl). Figure 2 shows the far-UV CD spectra obtained in phosphate buffer with no added salt for each fragment separately, the sum of the spectra of the two fragments, and the spectrum of the mixture of the two fragments.

The spectra of the separate fragments indicate largely unstructured polypeptides, while there is a distinct spectral change when comparing the spectrum of the mixture with the sum of the fragments. The spectrum of the mixture is highly similar to that of intact PGB1-QDD, but with lower intensity, showing that the two fragments form a complex with similar structure as intact PGB1-QDD. Similar results are obtained at all salt concentrations, showing that the two fragments interact with relatively high affinity under all these conditions. Similar results have been reported for the wild type protein in 50 mM sodium phosphate buffers [25]. ^1^H-NMR spectra for the mixture of fragments show several characteristics in common with intact PGB1-QDD, which are not seen in spectra that are of the free fragments (data not shown), again in accordance with the wild type [25].

### 2.2. Salt-dependent residual structure in C16

**Figure 3.**
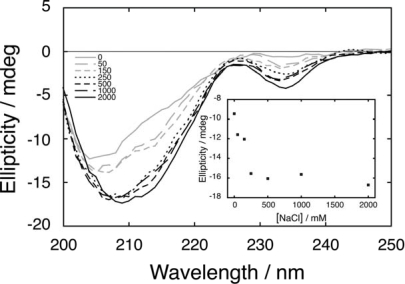
Far UV-CD spectra of C16 in different concentration of salt (grey solid line 0 mM, grey dashes 50 mM, short grey dashes 150 mM, black dotted 250 mM, short black dashes 500 mM, black dashes 1,000 mM and solid black line 2,000 mM NaCl). All samples contain 584 μM C16 in 5 mM sodium phosphate buffer pH 5.5 at 278 K. The inset shows the mean ellipticity from 207 to 214 nm as a function of salt concentration.

Some residual structure appears to be present in C16 fragment even when the N41-QDD fragment is absent. The spectra for the isolated C16 fragment as a function of salt concentration are shown in Figure 3. In the absence of added salt the spectrum reveals little structure. Minima appear at 206 nm and 235 nm and deepen with increasing salt. The deepening and shifting of the broad minimum around 206 most likely reflects structuring of the β-hairpin when salt is added, while changes in the cotton-effect region for aromatic side chains around 235 nm likely reports on structural changes around the Trp side chain. These data indicate that the C16 fragment may be partially folded as a β-hairpin and the fraction folded increases with increasing salt concentration which screens electrostatic repulsion with the fragment (net charge -3 over 16 residues). The effect of salt seems to level off at around 250 mM NaCl (inset, Figure 3), giving a Debye length of 6 Å.

### 2.3. Temperature dependence of CD spectra for fragment mixtures

The temperature dependence of reconstitution was studied through thermal denaturation of titration mixtures with constant concentration of C16 and increasing concentration fragment corresponding to N41-QDD. At each one of the four salt concentrations studied these data contain the dependence of the CD signal on the molar ratio of N41-QDD over C16 at each temperature. The same data also reveal at each salt concentration the temperature dependence of the CD signal at each molar ratio.

### 2.4. Affinity between N41-QDD and C16

**Figure 4.**
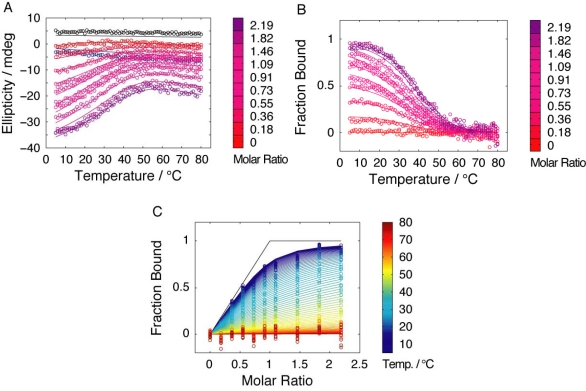
Titration of C16 with N41-QDD at 150 mM added NaCl as monitored by the CD signal at 218 nm. **(A)** Raw data (circles) together with globally fitted model as solid lines. **(B)** Fraction of C16 in complex with N41-QDD as function of temperature. **(C)** Fraction of C16 in complex with N41-QDD as function of the molar ratio. In (A) and (B), the colors represent molar ratio is represented by the color bar and with the background signal in black and isolated N41-QDD in blue. In (C) the colors represent temperature.

The affinity between N41-QDD and C16 was deduced from the CD signal dependence on the concentration of N41-QDD while keeping C16 at fixed concentration. Data from these titration experiments was first fitted using a simple 1:1 binding model (see Experimental section equations 1 – 4 and 10). Subsequently, we fitted the data using an extended model that assumes a much higher affinity of prefolded compared to unfolded C16 fragment for the N-terminal fragment (equations 5 – 9 and 10). All data were fitted globally with the same baselines and signal contribution of the equilibrium species for all four salt concentrations as well as a single free energy function of temperature to describe how binding constants vary with temperature for each salt concentration (equation 10). Raw data at 150 mM NaCl and the curve fitted globally are shown in Figure 4. The signal change as function of temperature for the free C16 fragment (at molar ratio of 0 in Figures 4A, and 4B), again shows that residual structure is present in this fragment even when the N41-QDD fragment is absent.

**Figure 5.**
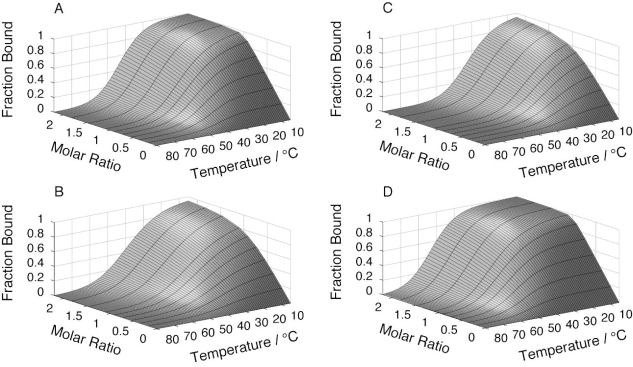
Globally fitted model surfaces of fraction C16 in complex with N41-QDD from titration experiments as function of temperature and molar ratio at (A) no added salt, (B) 150 mM, (C) 500 mM and (D) 2 M NaCl. The mesh lines represent locations of data points.

The fraction of C16 in complex with N41-QDD as obtained from the fit for each of the four salt concentrations is represented as a surface in Figure 5. The effective association constants obtained for the complex at 278 and 298 K are shown in Table 1. The smaller and shallower plateau observed at 150 mM and 500 mM NaCl (Figure 5B and C) indicates a lower overall stability of the complex for these conditions as compared to no added salt and 2 M NaCl. This trend is present over the whole temperature range where reliable data could be obtained.

### 2.5. Prefolding of C16 favors complex formation

The partial folding of the isolated C16 fragment of PGB1 in pure water as a β-hairpin [31] has stimulated considerable experimental and theoretical work towards understanding its folding and stability. With 16 amino acids, it is one of the smallest peptides that folds into a defined structure and thus is a good model for formation of β-hairpin and β-sheet structure. Previous studies report quite varied stability for the β-hairpin. Using thoroughly desalted samples of this fragment in the current study has allowed us to observe a salt dependence of the folding of the β-hairpin structure, and the amount of structure increases with the salt concentration (Figure 3). This stabilizing effect of salt likely derives from the very high charge density of the fragment, five charged amino acids, and four of them carry negative charges, out of just 16 residues. There will be less electrostatic repulsion in the folded β-hairpin in the presence of salt due to screening. The impact of β-hairpin folding on reconstitution has previously been investigated by introduction of a disulfide bond in the C16 fragment to favour the β-hairpin, which resulted in one order of magnitude higher affinity [37]. The maximum effect of salt found in the present study is an approximately 50-fold affinity enhancement going from medium to high salt concentration, corroborating the effect of preformed structure on the association constant.

### 2.6. Comparable salt effects on assembly and folding equilibria

**Figure 6.**
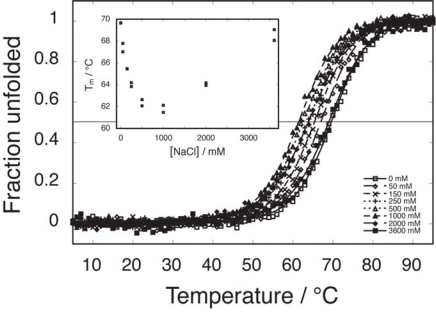
Thermal denaturation of intact PGB1-QDD in 5 mM sodium phosphate buffer pH 5.5 as a function of added salt monitored by CD at 218 nm. Fitted T_m_ values are shown in inset.

To compare the effects of salt on the binding affinity between the fragments with the effects on stability of the intact protein we performed a number of thermal melts of intact PGB1. The raw data, fits and the fitted T_m_ values are shown in Figure 6. In several other cases, a correlation has been shown between effects on protein stability towards denaturation and effects on the affinity between two fragments that reconstitute the protein [6,7,10]. The same salt effects as for the reconstitution affinity are seen for the stability of the intact protein with lower stability intermediate NaCl compared to no added or high salt. This similarity in salt dependence of reconstitution and folding further confirm the interrelation between the two processes.

For intact PGB1-QDD, the lower stability at intermediate compared to high or no added salt could be due to uneven shielding of favourable and unfavourable electrostatic interactions in our model protein. Similar to stability of the intact protein, the overall salt dependence of the reconstitution found here, with lower affinity at 150 mM and 500 mM salt, can only be explained by a number of counteracting effects involving salt. On the one hand, increasing the salt concentration increases the stability of the β-hairpin as seen in the CD-spectra for the C16 peptide likely due to electrostatic shielding of unfavourable charge repulsions. For example shielding of the electrostatic repulsion between E42 and E56 and between the carboxy terminus and E42 would lead to higher stability for the hairpin. This yields an increase in the affinity due to the fact that the prefolded fraction of the C16 fragment will lose fewer degrees of conformational freedom upon reconstitution. On the other hand salt also has a direct negative effect on the association. This effect, especially seen at low concentrations, could be due to screening of attractive electrostatic interactions between the two fragments, or to an effect of non-Coulombic character. Because of the long-range nature of electrostatic effects and the large number of charges in PGB1, it is difficult to isolate exactly which attractive electrostatic interactions could be behind this effect. Possible attractive interactions that may be reduced by shielding are between D40 and the N-terminus of the C16 fragment, between K31 and E42, and between K10 and the C-terminus. Thus, the fragmentation complementation approach here reveals that shielding of electrostatic interactions in PGB1 has two opposing effects, as previously observed in the intact protein [38,39].

## 3. Experimental Section

### 3.1. Cloning, expression and purification of PGB1 fragments

The PGB1-QDD fragments were cloned in the Intein plasmid system pTXb1 (Impact^™^CN; New England Biolabs, Beverly, MA, USA) from complementary oligonucleotides with the desired overhangs for ligation into the plasmid as cut with NdeI and SapI. The N-terminal fragment covers the sequence of PGB1-QDD residues 1 – 41 (N41-QDD). It contains Gly41 in order to increase the fragment yield, which depends on the identity of the C-terminal residue (Asp as found in position 40 being one of the worst, Impact ^™^CN manual). The C-terminal fragment corresponds to residues 41 – 56 (C16) of wt PGB1 sequence, which is the same as PGB1-QDD. Plasmids containing the confirmed sequences were transformed into *E. coli* strain ER2566. Overnight cultures (single colonies in LBmedium containing 50 μg/mL ampicillin) were diluted 1:100 into LB-medium containing 50 μg/mL ampicillin and subsequent production of the intein-fragment fusion protein was initiated through induction with 100 μg/mL IPTG at OD_600_ = 0.6. The harvested cells were sonicated, yielding the fusion protein in the supernatant. The sonicate was initially purified through ion-exchange chromatography using DEAE cellulose equilibrated with 10 mM Tris-HCl buffer, pH 7.5 and eluted using with the same buffer containing 0.5 M NaCl and the fusion protein was subsequently bound to a chitin column. The peptide fragments were cleaved off by 50 mM DTT in 10 mM Tris-HCl buffer at pH 7.5 with 1 mM EDTA, 0.5 M NaCl overnight or longer at 4 °C. The eluted fractions containing the peptide fragments were subjected to gel filtration to remove residual fusion protein, desalted and lyophilised. The identity and the purity of the peptide were verified through SDS-PAGE and amino acid analysis following acid hydrolysis (Biomedical Centre, Uppsala University).

For the subsequent measurements both the fragments and the intact protein were dissolved in 5 mM phosphate with 0, 0.15 or 2 M NaCl and the pH was adjusted to 5.5. The concentrations of the protein fragment stock solutions were determined by amino acid analysis after acid hydrolysis (Biomedical Centre, Uppsala University).

### 3.2. Expression and purification of intact PGB1-QDD

A plasmid preparation with verified DNA sequence was transformed into *E. coli* BL21 DES3 PLysS Star. Single colonies were used to inoculate overnight cultures of LB medium with 50 μg/mL ampicillin and 30 μg/mL chloramphenicol. The overnight cultures were diluted 1:100 in the day cultures of 500 mL each in 2.5 L baffled flasks. Protein production was induced by adding 0.4 mM isopropyl-*ß*-D-1-thiogalactoside at an OD_600_ of 0.6 – 0.8, and the culture was harvested by centrifugation 3 – 4 h later. The cell pellet was resuspended in H_2_O (120 mL for the pellet from a 5.4-liter culture), poured into 150 mL boiling buffer A (10 mM Tris/HCl and 1 mM ethylenedinitrilotetraacetic acid (EDTA), pH 7.5), heated to 80 °C and then directly cooled on ice. The solution was centrifuged at 15,000 g for 10 min, and the supernatant was rocked on ice with 80 mL DEAE cellulose. The cellulose was packed in a column, washed with buffer A, and eluted using a linear NaCl gradient from 0 to 400 mM in buffer A. Fractions containing PGB1-QDD were pooled and lyophilized, dissolved in 20 – 25 mL Millipore water (Millipore, Billerica, MA, USA), and separated on a 3.4 x 180 cm Sephadex (Sigma-Aldrich, St. Louis, MO, USA) G50 superfine gel filtration column using 50 mM ammonium acetate, pH 6.5, as running buffer. Fractions containing PGB1-QDD were pooled, lyophilized, and desalted on a Sephadex G25 superfine gel filtration column in water.

### 3.3. Circular dichroism spectroscopy

All circular dichromism (CD) experiments were carried out using a JASCO J-720 (JASCO, Tokyo, Japan) spectropolarimeter with a Peltier type thermostated cell holder. The reconstitution of the two fragments was followed between 5 and 80 °C for titration samples with different molar ratios. The CDsignal at 218 nm was recorded with a scan rate of 1 °C/min and a response of 8 s using 0.1 mm quartz cuvettes. For all the titration experiments temperature scans were performed for samples with 584 μM of the C-terminal (41 – 56) fragment and the concentration of the N-terminal (1 – 41) ranging from 0 to 1200 μM in nine steps. Far-UV CD spectra (250 – 190 nm) were recorded at 5 °C, with a scan rate of 20 nm/min with the same fragment concentrations as in the thermal scans. Samples were prepared by mixing the two protein fragment stock solutions and a NaCl-stock solution to achieve the desired peptide and NaCl concentrations. The thermal denaturation of intact PGB1 was followed between 5 and 95 °C using samples of 500 μM of intact PGB1.

### 3.4. ^1^H-NMR spectroscopy

^1^H-NMR spectra were recorded on a Varian Unity Plus 600 MHz spectrometer (Varian, Palo Alto, CA, USA) at 278 K. Each sample was dissolved in D_2_O with 5 mM phosphate buffer, pH 5.5 to a concentration of 500 μM

### 3.5. Binding model

Two models were used to fit the 2D simultaneous CD reconstitution and temperature denaturation data sets. A standard 1:1 binding model was first used. The reconstitution of PGB1 from the Nterminal (N) and C-terminal (C) fragments corresponds to the following equilibrium:

(1) $$N+C\overset{K_{A}}{⇄}NC$$

where the complex is denoted NC. With the assumption of ideal behaviour and the standard state at 1M, the equilibrium association constant, K_A_ and dissociation constant, K_D_ can be expressed as:

(2) $$K_{A}=\frac{1}{K_{D}}=\frac{\left[N\cdot C\right]}{\left[N][C\right]}$$

This can be expressed in terms of the total concentrations of the two fragments as:

(3) $$K_{A}=\frac{\left[N\cdot C\right]}{\left({\left[N\right]}_{tot}-[N\cdot C])\;({\left[C\right]}_{tot}-[N\cdot C]\right)}$$

and the complex concentration is given by:

(4) [N⋅C]=([N]tot+[C]tot+1KA)2−([N]tot+[C]tot+1KA)24−[N]tot[C]tot

Secondly, the data was also fitted using an extended model that also taking into account the folding equilibrium of the C-terminal β-hairpin fragment. For the folding of the C-terminal fragment, denoted C_U_ for the unfolded state and C_F_ for the folded state, we assume a two-state model. This extended model is composed by two coupled equilibria:

(5) $$\begin{array}{c} N+C_{F}\overset{K_{A}}{⇄}NC\\ C_{F}\overset{K_{U}}{⇄}C_{U} \end{array}$$

The equilibrium unfolding constant, given ideal behaviour of the solute, is:

(6) $$K_{U}=\frac{\left[C_{U}\right]}{\left[C_{F}\right]}$$

The 1:1 binding equilibrium in (5) was the same as (2) – (4) with the exception of C_F_ substituting C, yielding K_A_ as.

(7) $$K_{A}=\frac{\left[N\cdot C\right]}{\left({\left[N\right]}_{tot}-[N\cdot C])\;({\left[C\right]}_{tot}-[C_{U}]-[N\cdot C]\right)}=\frac{\left[N\cdot C]\;(1+K_{U}\right)}{\left({\left[N\right]}_{tot}-[N\cdot C])\;({\left[C\right]}_{tot}-[N\cdot C]\right)}$$

The complex concentration is given by:

(8) $$[N\cdot C]=\frac{\left({\left[N\right]}_{tot}+{\left[C\right]}_{tot}+\frac{1+K_{U}}{K_{A}}\right)}{2}-\sqrt{\frac{{\left({\left[N\right]}_{tot}+{\left[C\right]}_{tot}+\frac{1+K_{U}}{K_{A}}\right)}^{2}}{4}}-{\left[N\right]}_{tot}{\left[C\right]}_{tot}$$

where the effective association constant can be defined by comparing (4) and (9) as:

(9) $$K_{A,Eff}=\frac{K_{A}}{1+K_{U}}$$

The temperature dependence of the equilibrium constants in both models is:

(10) $$K=\text{exp}\left[\frac{\Delta G{}^{\circ}(T)}{RT}\right]=\text{exp}\left[\frac{\Delta H{}^{\circ}(T_{r})-T\Delta S{}^{\circ}(T_{r})+\Delta CP{}^{\circ}\left(T-T_{r}-\text{ln}\;\frac{T}{T_{r}}\right)}{RT}\right]$$

where T_r_ is a reference temperature. For the CD measurements, the total signal is assumed to be a linear combination of the signals each species.

### 3.6. Data Analysis

The data analysis was performed through non-linear least square analysis using scripts written in Matlab 7 (Mathworks, Natick, MA, USA). Binding model and the temperature dependence model explained above were fitted to all CD data globally through non-linear least-square regression analysis. The signal parameters are approximated as linear functions of the temperature. The slopes and intercepts of the signal values should be independent of the salt concentration and were therefore fitted as global parameters for all data. For the association equilibria, ΔCp was fitted as a global parameter while ΔCp was assumed to be close to zero for the unfolding equilibrium of the extended model. The enthalpy change and entropy change at the reference temperature T_r_ of 25 °C (10) wre fitted as global parameters within each salt concentration. For the unfolding equilibrium of the C16 fragment in the extended model, the parameter value of ΔH° (50 kJmol^−1^) from published DSC data [32] were used to further restrain the model in as the unfolding transition is not very well defined in the CD data because of the low stability of the β-hairpin. The parameter errors were determined using a non-parametric residual bootstrap method [40].

## 4. Conclusions

Using the fragment complementation approach we have here uncovered insights into the folding of PGB1 and provided linkages between the assembly of PGB1 subdomains and the protein’s stability behavior. Our results imply that folding of the PGB1 domain shifts with salt from a cooperative assembly of unstructured fragments to an assembly process involving pre-folded structures. This observation may be relevant for understanding the folding mechanism of PGB1 in physiological salt conditions.

## Figures and Tables

**Table 1.** Affinity between N41-QDD and C16 obtained by fitting to CD titration data using a 1:1 binding model (log K_A_) and an expanded three state model (log K_A,Eff_ equation 7).

[NaCl]	logK_A_ at 5 °C	logK_A_ at 25 °C	logK_A.Eff_ at 5 °C	logK_A.Eff_ at 25 °C
no added	5.4 ± 0.4	4.3 ± 0.2	5.4 ± 0.3	4.2 ± 0.2
150 mM	4.3 ± 0.1	3.7 ± 0.1	4.3 ± 0.1	3.6 ± 0.1
500 mM	4.4 ± 0.2	3.4 ± 0.2	4.3 ± 0.2	3.4 ± 0.1
2000 mM	6.0 ± 0.8	4.9 ± 0.4	5.7 ± 0.6	4.8 ± 0.4
